# A systematic review of the epidemiology of hepatitis A in Africa

**DOI:** 10.1186/s12879-019-4235-5

**Published:** 2019-07-22

**Authors:** Jenna Patterson, Leila Abdullahi, Gregory D. Hussey, Rudzani Muloiwa, Benjamin M. Kagina

**Affiliations:** 1 Vaccines for Africa Initiative, University of Cape Town, Room N2.09A, Werner Beit North, Health Sciences Campus, Anzio Road, Observatory, Cape Town, 7925 South Africa; 2 School of Public Health & Family Medicine, University of Cape Town, Cape Town, South Africa; 3 Save the Children International, Somaliland Country Office, Nairobi, Kenya; 4 Division of Medical Microbiology & Institute of Infectious Disease and Molecular Medicine, University of Cape Town, Cape Town, South Africa; 5 Department of Paediatrics & Child Health, Groote Schuur Hospital, The University of Cape Town, Cape Town, South Africa

**Keywords:** Hepatitis a virus, Africa, Seroprevalence, Epidemiology, Systematic review, Meta-analysis

## Abstract

**Background:**

Hepatitis A, caused by the hepatitis A virus (HAV), is a vaccine preventable disease. In Low and Middle-Income Countries (LMICs), poor hygiene and sanitation conditions are the main risk factors contributing to HAV infection. There have been, however, notable improvements in hygiene and sanitation conditions in many LMICs. As a result, there are studies showing a possible transition of some LMICs from high to intermediate HAV endemicity. The World Health Organization (WHO) recommends that countries should routinely collect, analyse and review local factors (including disease burden) to guide the development of hepatitis A vaccination programs. Up-to-date information on hepatitis A burden is, therefore, critical in aiding the development of country-specific recommendations on hepatitis A vaccination.

**Methods:**

We conducted a systematic review to present an up-to-date, comprehensive synthesis of hepatitis A epidemiological data in Africa.

**Results:**

The main results of this review include: 1) the reported HAV seroprevalence data suggests that Africa, as a whole, should not be considered as a high HAV endemic region; 2) the IgM anti-HAV seroprevalence data showed similar risk of acute hepatitis A infection among all age-groups; 3) South Africa could be experiencing a possible transition from high to intermediate HAV endemicity. The results of this review should be interpreted with caution as the reported data represents research work with significant sociocultural, economic and environmental diversity from 13 out of 54 African countries.

**Conclusions:**

Our findings show that priority should be given to collecting HAV seroprevalence data and re-assessing the current hepatitis A control strategies in Africa to prevent future disease outbreaks.

**Electronic supplementary material:**

The online version of this article (10.1186/s12879-019-4235-5) contains supplementary material, which is available to authorized users.

## Background

Hepatitis A is a vaccine preventable disease (VPD) caused by the hepatitis A virus (HAV). The hepatitis A virus is transmitted from person-to-person through the faecal-oral route primarily by ingestion of contaminated food or water and/or contact with infectious persons [1, 2]. Poor hygiene and sanitation pose the greatest risk for HAV infection, particularly in Low and Middle-Income Countries (LMICs) [3]. Infection with HAV causes an immune response which is assessed by measurement of specific antibodies: immunoglobulin class M (IgM) anti-HAV antibodies and immunoglobin class G (IgG) anti-HAV antibodies [4]. Anti-HAV IgM antibodies are detectable following acute infection and antibody titres usually decline to zero within 3–6 months [5, 6]. In contrast, anti-HAV IgG antibodies appear within 2–3 months after infection and persist for a long period of time conferring protective immunity against future infections [2]. A majority of hepatitis A seroprevalence studies, therefore, often report anti-HAV IgG and not anti-HAV IgM seroprevalence data.

Common clinical symptoms of hepatitis A infection include jaundice, fever, malaise, anorexia, nausea and abdominal discomfort [1, 4]. Infection with HAV in early childhood is thought to be largely asymptomatic and results in the development of lifelong protective immunity [4]. In contrast, infection with HAV after early childhood is associated with an increased risk of symptomatic, acute hepatitis A infection [1, 7, 8]. The case fatality rate associated with acute hepatitis A in children and adults < 50 years old ranges from 0.3 to 0.6%, while the case fatality rate in adults ≥50 years old ranges from 1.8 to 5.4% [9]. The high costs associated with management of acute hepatitis A are well appreciated by healthcare providers. Hepatitis A patients typically miss several weeks of work or school and the costs of supportive medical care can be substantial [4]. Therefore, vaccination against hepatitis A has been found to be cost-effective in many LMICs and should be prioritized in settings where hepatitis A is a public health concern [10]. Routine hepatitis A vaccination policies can only be developed based on up-to-date and high-quality contextual evidence that includes the burden of the disease.

The World Health Organization (WHO) describes the epidemiology of hepatitis A according to HAV endemicity levels [2]. Endemicity is measured by HAV seroprevalence; i.e. the proportion of people in the population with anti-HAV IgG antibodies [11]. The levels of HAV endemicity are classified by the WHO as follows: high (≥ 90% IgG seroprevalence by 10 years of age), intermediate (≥ 50% IgG seroprevalence by 15 years of age, < 90% IgG seroprevalence by 10 years of age), and low (≥ 50% IgG seroprevalence by 30 years of age, < 50% IgG seroprevalence by 15 years of age) [2]. The latest global review of HAV endemicity was published in 2010 and included epidemiological data from 1990 to 2005. The review classifies Africa as a high HAV endemic region [12]. Since 2005, many African countries have made significant improvements in water, sanitation and developments in socio-economic status (SES). These improvements are likely to cause changes in the average age of first exposure and infection with HAV as well as in the prevalence of acute hepatitis A. Recent hepatitis A studies conducted in Africa, though few and far between, suggest that some regions on the continent could be experiencing a transition in hepatitis A epidemiology. Our aim in this review is to provide an up-to-date synthesis of hepatitis A epidemiology in Africa.

## Rationale

The WHO does not recommend routine vaccination against hepatitis A in high endemic settings [2]. As of 2018, no African country included routine hepatitis A vaccination as part of its’ Expanded Programme on Immunisation (EPI). The WHO recommendation is that countries should routinely collect and review local factors and epidemiological data needed to guide the development of evidence-based recommendations on hepatitis A vaccination [2]. To the best of our knowledge, an up-to-date, comprehensive synthesis of hepatitis A epidemiological data in Africa is lacking. Though there have been several primary studies on hepatitis A epidemiology published since 2005 in Africa, the review team is not aware of any recent publication that has synthesised data from this setting [13–16]. The development of effective public health control strategies against hepatitis A require optimal characterisation of the disease epidemiology. Therefore, this systematic review aims to fill the existing knowledge gap to guide considerations of development of public health strategies to control hepatitis A in the region.

## Methods

### Objectives

To describe the epidemiology of hepatitis A in Africa.

#### Primary objectives

- To estimate the HAV seroprevalence (the prevalence of IgG anti-HAV antibodies) in Africa
- To estimate the prevalence of IgM anti-HAV antibodies
- To estimate the acute hepatitis A hospitalisation rate in Africa
- To estimate the acute hepatitis A case fatality rate in Africa

#### Secondary objective

- To assess the impact of co-morbidities on hepatitis A epidemiology in Africa

### Study eligibility criteria

Published and unpublished case-series, case-control, cross-sectional, cohort studies as well as randomised control trial (RCTs) and non-randomised control trial (nRCTs) in any language that reported the epidemiology of hepatitis A in children > 1 year of age as well as in adults in any African country were eligible for inclusion in this review. Studies were eligible for inclusion if they reported on any of the outcomes of this review, including seroprevalence of IgG anti-HAV antibody or prevalence of IgM HAV-antibody detection as well as hepatitis A disease incidence rates, hospitalisation rates, case fatality rates as well as co-infections.

### Search strategy

A combination of the following search terms (including the use of Medical Subject Headings (MESH)) was used: hepatitis A, acute hepatitis A, epidemiology, incidence, prevalence, morbidity, mortality, hospitalisation and case-fatality. An example of the search strategy as applied to PubMed is outlined in Table 1. The following electronic databases were searched for relevant published literature: EBSCOhost, MEDLINE via PubMed, ScienceDirect via SciVerse, Scopus via SciVerse, Ovid Discovery and Google Scholar. Grey literature was sourced by consulting with expert researchers in the field and by searching the following grey literature repositories: OpenUCT, OpenGrey, MEDNAR and CORE. The literature search was initially performed in February 2018 and updated in December 2018.

**Table 1:** Search Strategy for PUBMED

Query #	Search Query
#1	hepatitis A [MeSH Terms] OR hepatitis A [All Fields] OR acute hepatitis A [MeSH Terms] OR acute hepatitis A [All Fields]
#2	epidemiology [MeSH Terms] OR epidemiology [All Fields]
#3	incidence [MeSH Terms] or incidence [All Fields]
#4	prevalence [MeSH Terms] or prevalence [All Fields]
#5	morbidity [MeSH Terms] OR morbidity [All Fields] OR hospitalisation [MeSH Terms] OR hospitalisation [All Fields] OR hospitalization [MeSH Terms] or hospitalization [All Fields]
#6	mortality [MeSH Terms] OR mortality [All Fields] OR case-fatality [MeSH Terms] OR case-fatality [All Fields]
#7	Africa [MeSH Terms] OR Africa [All Fields] OR Algeria [All Fields] OR Angola [All Fields] OR Benin [All Fields] OR Botswana [All Fields] OR Burkina Faso [All Fields] OR Burundi [All Fields] OR Cabo Verde [All Fields] OR Cameroon [All Fields] OR Central African Republic [All Fields] OR Chad [All Fields] OR Comoros [All Fields] OR Congo [All Fields] OR Cote d’Ivoire [All Fields] OR Djibouti [All Fields] OR Egypt [All Fields] OR Equatorial Guinea [All Fields] OR Eritrea [All Fields] OR Ethiopia [All Fields] OR Gabon [All Fields] OR Gambia [All Fields] OR Ghana [All Fields] OR Guinea [All Fields] OR Guinea-Bissau [All Fields] OR Kenya [All Fields] OR Lesotho [All Fields] OR Liberia [All Fields] OR Libya [All Fields] OR Madagascar [All Fields] OR Malawi [All Fields] OR Mali [All Fields] OR Mauritania [All Fields] OR Mauritius [All Fields] OR Morocco [All Fields] OR Mozambique [All Fields] OR Namibia [All Fields] OR Niger [All Fields] OR Nigeria [All Fields] OR Rwanda [All Fields] OR Sao Tome and Principe [All Fields] OR Senegal [All Fields] OR Seychelles [All Fields] OR Sierra Leone [All Fields] OR Somalia [All Fields] OR South Africa [All Fields] OR South Sudan [All Fields] OR Sudan [All Fields] OR Swaziland [All Fields] OR Tanzania [All Fields] OR Togo [All Fields] OR Tunisia [All Fields] OR Uganda [All Fields] OR Zambia [All Fields] OR Zimbabwe [All Fields]
#8	2005 [PDAT]: 2018 [PDAT]
#9	#1 AND #2 AND #3 AND #4 AND #

Age of participants are included in search filter

*Abbreviations*: *MeSH* Medical Subject Heading, *PDAT* Publication date

### Data extraction

Study characteristics and outcomes of interests were extracted from the included studies on a pre-designed data extraction form by two independent reviewers (JP and LA). Prior to use by the two reviewers, the reliability of the extraction form was assessed by piloting 10 randomly selected articles that met the inclusion criteria. The study resolved any disagreements in data extraction through consensus in consultation with BMK. In cases where studies were not available in English, google translate was used to translate the article to English [17].

### Data synthesis and analysis

A random effects model was fitted to the study data as it includes estimates taken from a series of independently performed studies. Where heterogeneity between included studies was found to be low in meta-analyses (*I*^2^ < 75), pooled outcome measures were reported with 95% confidence intervals for each respective outcome. Where heterogeneity was found to be high in meta-analyses (*I*^2^ ≥ 75), narrative reporting was used to describe the prevalence ranges for each respective outcome.

### Risk of bias

Each included study was assessed for risk of bias and quality using the Hoy et al.*,* 2012 tool for observational studies [18, 19]. All risk of bias judgements were made by JP and LA. In case of disagreement in risk of bias and quality assessment, a final decision was made through consensus in consultation with BMK.

### Reporting of review

This systematic review was registered with PROSPERO (registration number CRD42017079730) and the results are reported using the Preferred Reporting Items for Systematic Reviews and Meta-analyses (PRISMA) guidelines checklist (Additional file 1) [20].

## Results

**Fig. 1:**
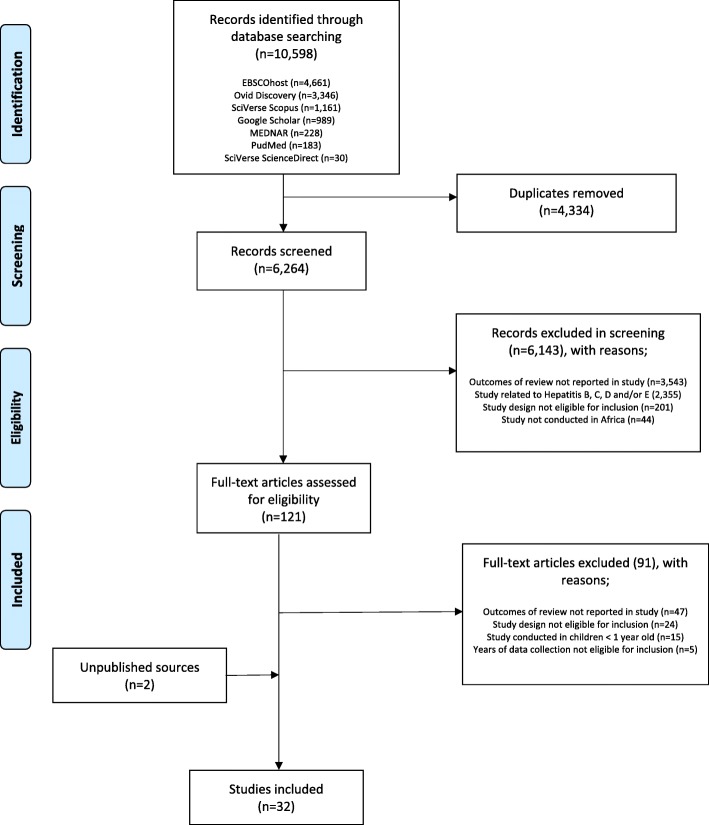
Flow diagram for selection of studies. PRISMA flow diagram of study selection process

**Fig. 2:**
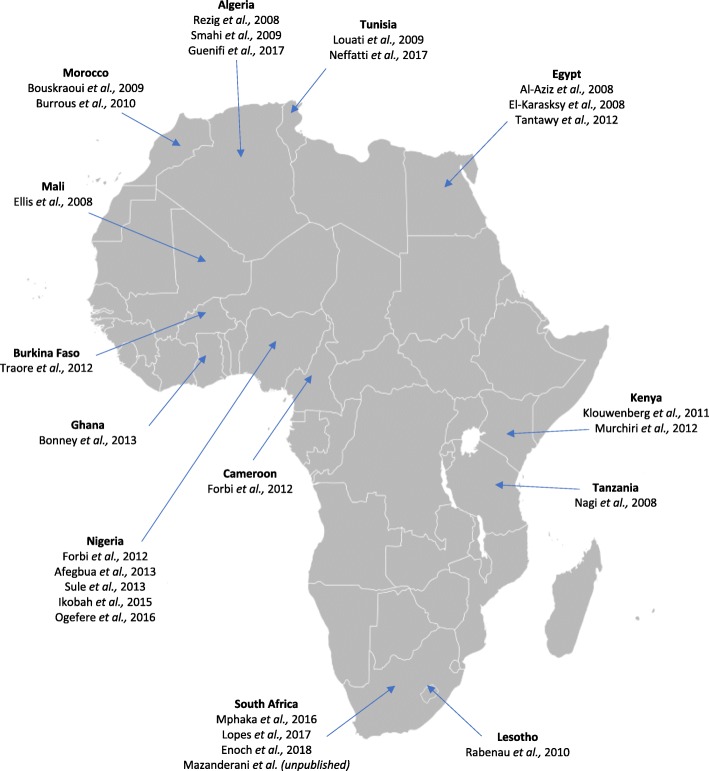
Map of included studies. Map of studies included in this systematic review. Displays the geographical location of 28 of 32 included studies (4 excluded studies report hepatitis A data from Africa generally). Map adapted from Wikimedia Commons (https://commons.wikimedia.org/wiki/File:BlankMap-Africa.svg)

The initial database searches yielded 10,598 records, from which 4,334 duplicates were removed. No additional records were found when the search was updated in December 2018. A further 6,264 records were excluded following the screening of titles and abstracts (Fig. 1). The full-text of the remaining 121 records were screened, from which 30 records met the final inclusion criteria. A further two unpublished studies at the time of the search were obtained through personal communication with hepatitis A researchers [11, 21]. Since the time of reciept of these studies, they have since been published. Therefore, a total of 32 studies were included in this review. The included studies were conducted in 13 African countries, a majority of these being from the North, West and Southern regions of the continent (Fig. 2). Figure 2 displays the geographic location of 27 of the included studies conducted on the African continent. Five of the 32 included studies (not shown in Fig. 2) reported hepatitis A data from expatriate communities (adults and children) from the African continent, living in Europe and North America [22–26].

Twenty-three of the included studies were cross-sectional studies (Table 2). A majority of the included studies were conducted in the public healthcare sectors of lower-middle income countries. Of the 32 included studies, 17 provided data on anti-HAV IgG alone (referred to hereon as HAV seroprevalence), 11 provided data on anti-HAV IgM alone (referred to hereon as IgM anti-HAV seroprevalence) and 4 studies provided data together for IgG anti-HAV and IgM anti-HAV seroprevalence. Our analyses categorize the included studies according to the population age-groups [children & adolescents (1 to 18 years of age), adults (> 18 years of age) and all ages (1 to 99 years of age)], of which children and adolescent populations were most commonly reported on (56% of included studies). Measurement of the anti-HAV antibodies was assessed using ELISA assays for both IgG and IgM positivity in all studies. Real time PCR (RT-PCR) was used in 4 studies, in addition to the ELISA assay (Table 3). Details on the assay detection limits were missing from all included studies.

**Table 2:** Characteristics of studies included in the review

Author, Year (Citation)	Study Design	Year(s) of Data Collection	Country	Population	Sample Size (n)	Outcome Measures	Study Objective
Abdulla et al., 2010 [22]	Cross-sectional	2006 to 2008	General Africa	Children & adolescents	29	IgG	To determine the prevalence of acute hepatitis A virus infection and immunity among internationally adopted children
Afegbua et al., 2013 [27]	Cross-sectional	2009	Nigeria	Children & adolescents	403	IgG	To determine seroprevalence of HAV among schoolchildren and adolescents in Kaduna State and identify factors associated with seropositivity
Al-Aziz et al., 2008 [28]	Cohort	2008	Egypt	Children & adolescents	296	IgG	To determine the seroprevalence of HAV antibodies among group of children
Blanchi et al., 2014 [23]	Cohort	2009 to 2012	General Africa	Children	146	IgM	To describe infectious diseases in internationally adopted children
Bonney et al., 2013 [29]	Cross-sectional	2008 to 2011	Ghana	All ages	285	IgM	To determine if viral hemorrhagic fevers and viral hepatitides contribute to hospital morbidity in the Central and Northern parts of Ghana
Bouskraoui et al., 2009 [30]	Cross-sectional	2005 to 2006	Morrocco	Children & adolescents	150	IgG	To assess the prevalence of viral hepatitis A infection in febrile icteric children and to examine the main risk factors of transmission
Burrous et al., 2010 [31]	Cross-sectional	2006 to 2008	Morrocco	Children & adolescents	129	IgM	To assess the prevalence of viral hepatitis A infection in febrile icteric children and to examine the main risk factors of transmission
El-Karasksy et al., 2008 [32]	Cohort	2005	Egypt	Children & adolescents	172	IgG	To determine the prevalence of anti-hepatitis A virus antibodies among 172 children with chronic liver disease
Ellis et al., 2008 [33]	Cohort	2008	Mali	Children	36	IgM	Phase 1 study in Malian children of the blood stage malaria vaccine
Enoch et al., 2019 [21]	Cross-sectional	2009 to 2015	South Africa	Children	482	IgG	To determine the seroprevalence of hepatitis A infection in the Western Cape Province of South Africa
Forbi et al., 2012 [34]	Cohort	2012	Cameroon	Children	78	IgM	To undertake genetic analysis of the hepatitis A virus associated with cases of acute diarrhea among children under five in Cameroon
Forbi et al., 2012_2 [35]	Cross-sectional	2006	Nigeria	Adults	114	IgM	To investigate HAV strains among apparently healthy adult Nigerian subjects
Guenifi et al., 2017 [36]	Cross-sectional	2010 to 2011	Algeria	Children	1061	IgG	To estimate the seroprevalence of hepatitis A virus infection in the district of Setif
Ikobah et al., 2015 [37]	Cross-sectional	2012	Nigeria	Children & adolescents	406	IgG	To determine the seroprevalence and predictors of viral hepatitis A in children aged 1 to 18 years
Jablonka et al., 2017 [38]	Cross-sectional	2015	General Africa	All ages	55	IgG	To determine the seroprevalence of anti-HAV IgG in refugees in Germany
Klouwenberg et al., 2011 [39]	Cohort	2011	Kenya	Children	222	IgM	To determine the temporal pattern of a co-infection of *P. falciparum* malaria and acute HAV in a cohort of Kenyan children under the age of five
Lopes et al., 2017 [40]	Cross-sectional	2015	South Africa	All ages	300	IgG	To determine the seroprevalence of HAV and HEV antibodies in blood donors giving at the Western Province Blood Transfusion Service
Louati et al., 2009 [41]	Cross-sectional	2007	Tunisia	Adults	376	IgG	To assess hepatitis A virus seroprevalence in blood donors from South Tunisia in two periods; 200 and 2007
Majori et al., 2008 [26]	Cross-sectional	2008	General Africa	All ages	182	IgG & IgM	To assess the seroprevalence of viral hepatitis infections in sub-Saharan immigrants living in Italy
Mazanderani et al., 2018 [11]	Cross-sectional	2005 to 2015	South Africa	All ages	501083	IgG & IgM	To assess seroprevalence rates among specimens tested for HAV serology within South Africa’s public health sector
Mphaka et al., 2016 [42]	Cross-sectional	2016	South Africa	Children & adolescents	46	IgM	To respond to an increase in blood samples testing positive for HAV IgM
Murchiri et al., 2012 [43]	Cross-sectional	2007 to 2008	Kenya	Adults	100	IgM	To determine seroprevalence of HAV, HBV HCV and HEV among patients with acute hepatitis in Nairobi Kenya
Nagu et al., 2008 [44]	Cross-sectional	2006	Tanzania	Adults	260	IgM	To determine the prevalence and predictors of viral hepatitis co-infection among HIV-infected individuals presenting at the HIV care and treatment clinics in the country
Neffatti et al., 2017 [45]	Cross-sectional	2014 to 2015	Tunisia	Adults	216	IgG	To supplement lacking data on hepatitis A and E from rural areas of South Tunisia
Ogefere et al., 2016 [46]	Cross-sectional	2016	Nigeria	All ages	200	IgM	To determine the seroprevalence of anti-HAV IgM in an at-risk population in Benin City and to identify the social, demographic and other risk factors
Raabe et al., 2014 [24]	Cross-sectional	2014	General Africa	Children	656	IgM	To assess the need to recommend routine HAV vaccination in internationally adopted children
Rabenau et al., 2010 [47]	Cohort	2007	Lesotho	Adults	205	IgG	To screen international adoptees for acute HAV infection
Rezig et al., 2008 [48]	Cross-sectional	2008	Algeria	Children & adolescents	3357	IgG	To assess the seroprevalence of coinfecting viruses in a cohort of 205 HIV-infected individuals
Smahi et al., 2009 [49]	Cross-sectional	2006	Algeria	Children	252	IgG	To determine the seroprevalence of hepatitis A and E infections
Sule et al., 2013 [50]	Cross-sectional	2010 to 2011	Nigeria	All ages	91	IgG	To determine the prevalence of anti-hepatitis A virus IgG antibody and associated factors among residents of Osogbo
Tantawy et al., 2012 [51]	Case-control	2009 to 2010	Egypt	Children & adolescents	182	IgG	To evaluate the seroprevalence of hepatitis A in Egyptian patients with haemophilia A
Traore et al., 2012	Cross-sectional	2010 to 2012	Burkina Faso	Adults	91	IgG & IgM	To assess the seroprevalence of antibodies to both HAV and HEV in central Burkina Faso in the absence of a recorded hepatitis epidemic

*Abbreviations*: *HAV* Hepatitis A virus, *IgG* Immunoglobin class G, *HBV* Hepatitis B virus, *HCV* Hepatitis C virus, *HEV* Hepatitis E virus

**Table 3:** Assays used in included studies

Author, Year	Assay	Brand
Abdulla et al., 2010 [22]	ELISA	DiaSorin
Afegbua et al., 2013 [27]	ELISA	Asia-lion Bitechnology
Al-Aziz et al., 2008 [28]	ELISA	DiaSorin
Blanchi et al., 2014 [23]	Serology	NR
Bonney et al., 2013 [29]	RT-PCR	RealStar
Bouskraoui et al., 2009 [30]	ELISA	NR
Burrous et al., 2010 [31]	ELISA	DiaSorin
El-Karasksy et al., 2008 [32]	ELISA	DiaSorin
Ellis et al., 2008 [33]	Serology & ALT levels	NR
Enoch et al., 2019 [21]	ELISA	Siemens
Forbi et al., 2012 [34]	RT-PCR	Applied Biosystems
Forbi et al., 2012_2 [35]	RT-PCR	NR
Guenifi et al., 2017 [36]	ELISA	Roche
Ikobah et al., 2015 [37]	EIA	DRG International Inc.
Jablonka et al., 2017 [38]	ELISA	Abbott ARC
Klouwenberg et al., 2011 [39]	ELISA	BioChain
Lopes et al., 2017 [40]	ELISA	Abbott ARC
Louati et al., 2009 [41]	ELISA	DiaSorin
Majori et al., 2008 [26]	ELISA	Abbott ARC
Mazanderani et al., 2018 [11]	Serology	NR
Mphaka et al., 2016 [42]	Serology	NR
Murchiri et al., 2012 [43]	ELISA	NR
Nagu et al., 2008 [44]	ELISA	Adaltis
Neffatti et al., 2017 [45]	RT-PCR	Wantani
Ogefere et al., 2016 [46]	Serology	Qingdao High-top Biotech
Raabe et al., 2014 [24]	Serology	N/A
Rabenau et al., 2010 [47]	ELISA	AxSYM MEIA
Rezig et al., 2008 [48]	ELISA	Bio-Rad
Smahi et al., 2009 [49]	Serology	NR
Sule et al., 2013 [50]	ELISA	DiaSorin
Tantawy et al., 2012 [51]	ELISA	DiaSorin
Traore et al., 2012	ELISA	DiaSorin

*Abbreviations*: *NR* Not reported, *ELISA* Enzyme-linked immunosorbent assay, *RT-PCR* Reverse transcription polymerase chain reaction, *EIA* Competitive enzyme immunoassay, *ALT* Alanine aminotransferase

### HAV seroprevalence in Africa from 2008 to 2018

**Fig. 3:**
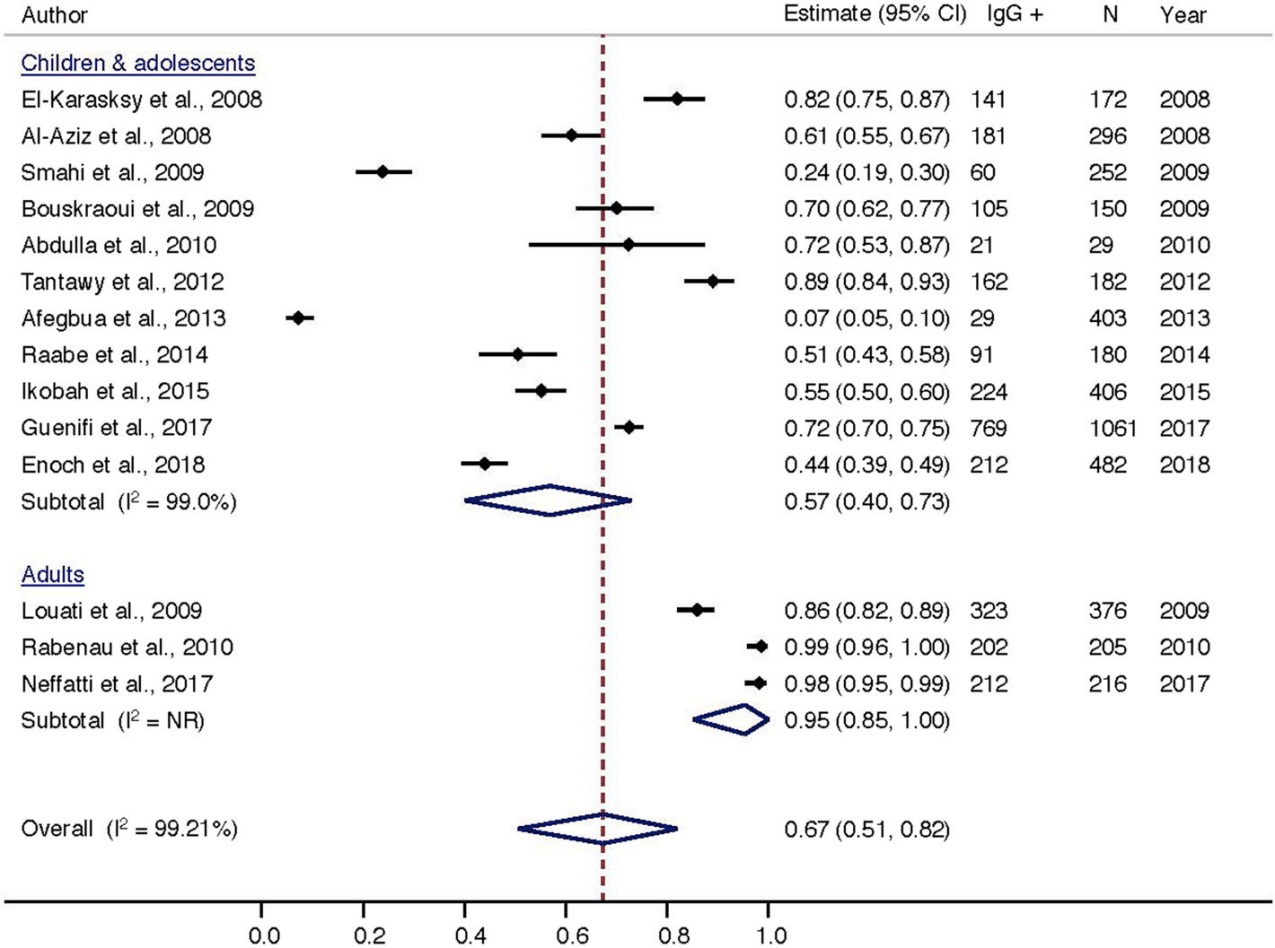
HAV seroprevalence by population group in africa, 2008-2018

Heterogeneity was high (*I*^*2*^ = 99.21%) among the 15 studies pooled for analysis of IgG seroprevalence in all age groups. This was not surprising considering the diversity of the included studies, thus we categorized the analysis of HAV seroprevalence by age-groups (Fig. 3). The estimated average of the reported HAV seroprevalence for children and adolescents among included studies was 57.0% (ES = 0.57; 95% CI = 0.40, 0.73) as compared to compared to 95.0% (ES = 0.98; 95% CI = 0.85, 1.00) for adults.

**Fig. 4:**
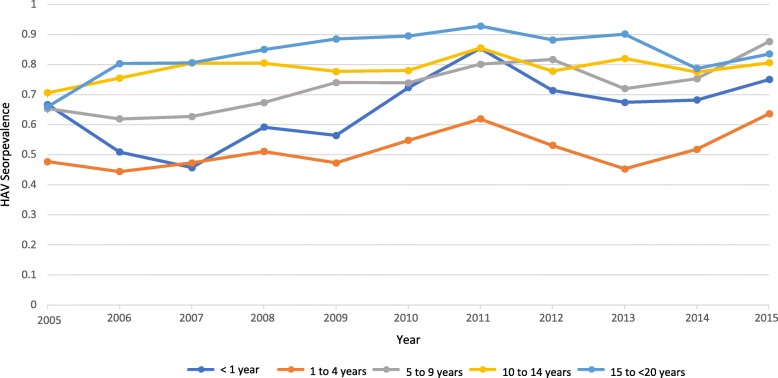
HAV seroprevalence estimates by age-group in South Africa, 2005-2015

Data reported by Mazanderani et al.*,* (2018) presented a unique opportunity to further explore of HAV seroprevalence by age-groups in South Africa from 2005 to 2015 (Fig. 4). The data displayed in Fig. 4 shows that HAV seroprevalence for children, adolescents < 15 years old remained below 90% for any given year between 2005 and 2015. Additionally, Fig. 4 shows that HAV seroprevalence for adolescents ≥ 15 and adults < 20 reduced from its highest in 2011 (92.8%) to 83.5% in 2015.

### IgM anti-HAV seroprevalence in Africa from 2008 to 2018

**Fig. 5:**
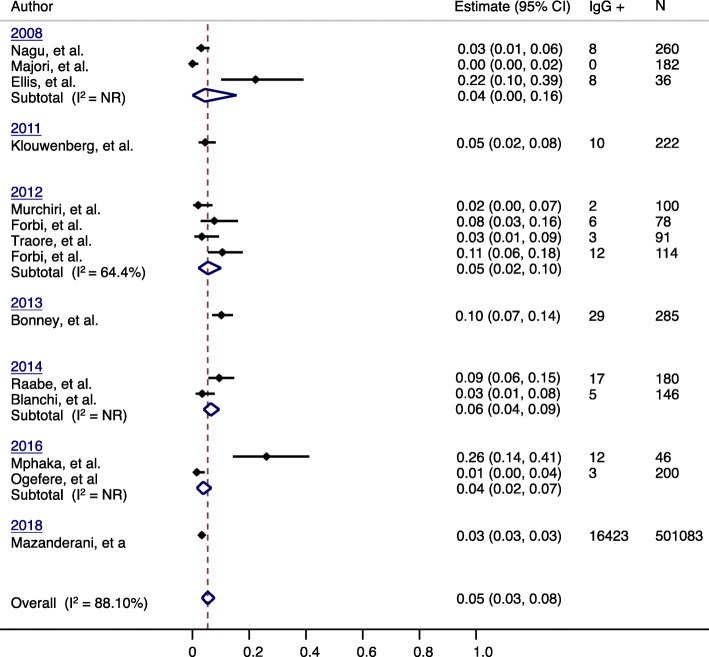
IgM anti-HAV seroprevalence in Africa, 2008-2018

We have used IgM anti-HAV seroprevalence as a marker for acute hepatitis A infection in this review [52]. Pooled acute hepatitis A prevalence for 2008 to 2018 showed high heterogeneity (*I*^*2*^ = 98.1%) (Fig. 5**).** An outlier in the data (Burros et al., 2010) reported acute hepatitis A prevalence in a population of febrile icteric children [91.0% (ES = 0.91; 95% CI = 0.85, 0.96)] and removed from the analysis. With removal of the outlier from the dataset, the average annual acute hepatitis A prevalence was reported to be approximately 5.0% (ES = 0.05; 95% CI = 0.03, 0.08).

**Fig. 6:**
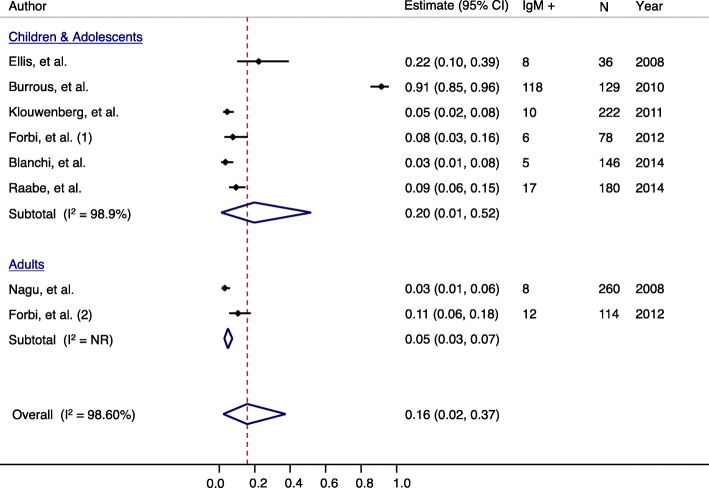
IgM HAV seroprevalence by population group in Africa, 2008-2014

We further explored the age-related risk of acute hepatitis A infection in Africa. When assessing IgM anti-HAV seroprevalence by age-group, the heterogeneity between studies was found to be relatively low (*I*^2^ = 74.73) (Fig. 6). The estimated average IgM anti-HAV seroprevalence for children and adolescents among included studies was 7.0% (ES = 0.07; 95% = 0.04, 0.12) (Fig. 6). The estimated average IgM anti-HAV seroprevalence for adults among included studies was 5.0% (ES = 0.05; 95% = 0.03, 0.07) (Fig. 6). The similarity in the estimated IgM anti-HAV seroprevalences among children, adolescents and adults is not expected in a high HAV endemic region such as Africa.

### IgM anti-HAV seroprevalence in South Africa

**Fig. 7:**
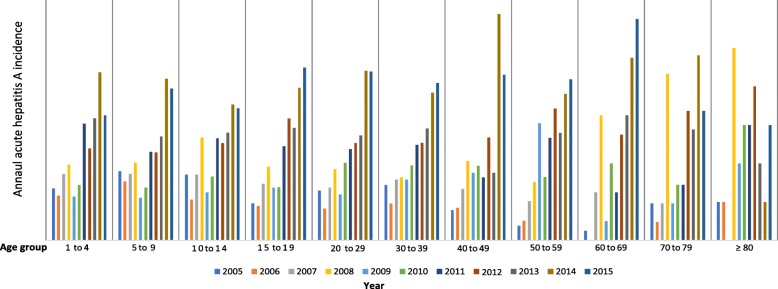
IgM anti-HAV seroprevalence estimates by age-group in South Africa, 2005-2015

Data reported by Mazanderani et al.*,* (2018) allowed us to further explore age-related IgM anti-HAV seroprevalence in South Africa, a country with no routine hepatitis A vaccination [11]. Figure 7 shows the annual IgM anti-HAV seroprevalence by age-group between 2005 and 2015 in South Africa, in which the overall IgM anti-HAV seroprevalence was found to be highest in children < 15 years of age. Acute hepatitis A infection rates over the decade for age groups < 10 years of age and 10 to 14 years of age were approximately 16.5 and 15.0%, respectively. The prevalence of acute hepatitis A in South Africa appeared to increase for all reported age-groups between 2005 and 2015.

### Methodological quality

For each included study, risk of bias and quality assessments were conducted using the Hoy et al.*,* risk of bias tool that examines internal and external validity of observation studies. Studies were judged as having ‘low risk’ if scored 8–10, ‘moderate risk’ if scored 5–7 and ‘high risk’ if scored 0–5. Scores were assigned by two (JP and LA) reviewers and the reasons for the assigned score was provided (Table 4). The scores assigned by the two reviewers we then compared. Where the assigned score made by JP and LA differed, these differences were resolved through consensus in consultation with BMK. For any score below 10, a descriptive summary of the information that influenced our judgments was provided. Majority of the studies were scored either 10 or 8 due to one or a combination of the following reasons: 1) selection of the research location was not justified; 2) Selection of study participants was not generalizable to the entire population; 3) Selection bias may be present.

**Table 4:** Risk of Bias assessment for included studies

Author, Year	Risk of Bias	Hoy et al. tool Score	Score Description
Abdulla et al., 2010 [22]	Low	10	
Afegbua et al., 2013 [27]	Low	8	1) Selection of research location was convenience and not justified as generalizable to entire population; 2) No description of how survey was conducted is given
Al-Aziz et al., 2008 [28]	Low	9	1) Selection of research location was convenience and not justified as generalizable to entire population
Blanchi et al., 2014 [23]	Low	10	
Bonney et al., 2013 [29]	Low	9	1) Selection of research location was convenience and not justified as generalizable to entire population
Bouskraoui et al., 2009 [30]	Low	10	
Burrous et al., 2010 [31]	Low	10	
El-Karasksy et al., 2008 [32]	Low	9	1) Selection of research location was convenience and not justified as generalizable to entire population
Ellis et al., 2008 [33]	Low	10	
Enoch et al., 2019 [21]	Low	10	
Forbi et al., 2012 [34]	Low	9	1) Selection of research location was convenience and not justified as generalizable to entire population
Forbi et al., 2012_2 [35]	Low	9	1) Selection of research population was not justified as generalizable to entire population
Guenifi et al., 2017 [36]	Low	9	1) Selection of research population was not justified as generalizable to entire population
Ikobah et al., 2015 [37]	Low	9	1) Selection of total anti-HAV antibody testing may confound results
Jablonka et al., 2017 [38]	Low	10	
Klouwenberg et al., 2011 [39]	Low	9	1) Selection of research population was not justified as generalizable to entire population
Lopes et al., 2017 [40]	Low	9	1) Years of data collection not described in publication
Louati et al., 2009 [41]	Low	10	
Majori et al., 2008 [26]	Low	9	1) Selection of research population was not justified as generalizable to entire population
Mazanderani et al., 2018 [11]	Low	10	
Mphaka et al., 2016 [42]	Low	8	1) Selection of research population was not justified as generalizable to entire population; 2) No random selection or census undertaken
Murchiri et al., 2012 [43]	Low	8	1) Purposive sampling leads to selection bias; 2) Selection of research population was not justified as generalizable to entire population
Nagu et al., 2008 [44]	Low	9	1) Selection of research population was not justified as generalizable to entire population
Neffatti et al., 2017 [45]	Low	10	
Ogefere et al., 2016 [46]	Low	9	1) Sampling method may have led to selection bias
Raabe et al., 2014 [24]	Low	9	1) Selection of research population was not justified as generalizable to entire population
Rabenau et al., 2010 [47]	Low	9	1) Selection of research population was not justified as generalizable to entire population
Rezig et al., 2008 (55)	Low	10	
Smahi et al., 2009 (56)	Low	10	
Sule et al., 2013 (57)	Low	9	1) Selection of research population was not justified as generalizable to entire population
Tantawy et al., 2012 (58)	Low	10	
Traore et al., 2012 (59)	Low	9	1) Selection of research location was convenience and not justified as generalizable to entire population

## Discussion

This systematic review evaluated the epidemiology of hepatitis A in participants > 1 year of age in Africa. The main findings of the review include: 1) the reported HAV seroprevalence data suggests that Africa, as a whole, should not be considered as a high HAV endemic region; 2) the IgM anti-HAV seroprevalence data showed similar risk of acute hepatitis A infection among all age-groups; 3) South Africa could be experiencing a possible transition from high to intermediate HAV endemicity. The results of this review were limited due to lack of detailed age-grouped data from the included studies. Additionally, no included review reported data on the hospitalisation and case fatality rates or co-morbidities occurring with acute hepatitis A which did not allow for the objectives of the paper to be met fully.

Only 13 (24%) out of 54 countries in Africa contributed to the data synthesized in this review. Furthermore, the data included in this review was collected mainly in hospital settings as opposed to from community surveys. A recent study on trends of childhood immunisation research in Africa reported lack of hepatitis A research on the continent [53]. Based on these findings, we believe that more up-to-date research on hepatitis A epidemiology in Africa is needed and will be critical to generate evidence needed to re-think hepatitis A control strategies in the region.

Although limited, the HAV seroprevalence data in this review appear to meet the WHO’s definition of intermediate HAV endemic setting (< 90% IgG seroprevalence by 10 years of age and ≥ 50% IgG seroprevalence by 15 years of age) [54]. The reported HAV seroprevalence estimates for children and adolescents age-groups indicate that the presumed “natural immunisation” during the early childhood is not sufficient to imply high HAV endemicity for the entire continent. Secondly, the reported similarity of IgM anti-HAV seroprevalence among children and adolescents compared with adults was a surprising finding as we expected lower IgM anti-HAV seroprevalence in adults than children due to prior exposure in a high endemic setting. A recent study in China and conducted in a setting undergoing a hepatitis A epidemiological transition, adults aged 20 years and older showed higher disease incidence than children [55]. Thus, our findings corroborate the notion of a HAV epidemiological transition in the African region.

Current global recommendations on hepatitis A vaccination appear to take African countries as homogeneous settings [54]. Our review results showed a large spread in HAV seroprevalence rates as well as IgM anti-HAV seroprevalence rates across the continent. This indicates the heterogeneity of hepatitis A epidemiology, and highly likely, the epidemiology of other VPDs among African countries. For example, in South Africa where comprehensive dataset was available, we reported an increase in IgM anti-HAV seropositivity among all age groups from 2005 to 2015. These results indicate that South Africa is most likely transitioning from high to intermediate endemicity. Previous classifications of South Africa as a high endemic region have been based on limited data published between 1986 and 2002 [56]. This data showed variable HAV seroprevalence rates that were dependent on SES. High HAV seropositivity rates were reported in low SES groups, while high SES groups that were less represented in the data showed low HAV seropositivity rates. Given this and the gradual social economic improvements in South Africa since the collapse of apartheid, it is likely that the HAV epidemiological transition in South Africa has been taking place even before 2005. It would be irrational to extrapolate findings from South Africa to all other African countries as Hepatitis A epidemiology is highly influenced by economic as well as healthcare developments [57]. Our findings suggest that African countries with similar SES developments as South Africa should prioritize generating evidence to guide recommendations on introducing routine immunisation against the disease.

The results of this review must be interpreted with caution due to several limitations. Firstly, the included studies have significant sociocultural, economic and environmental diversity. Secondly, due to the fact that only 13 of 54 countries in Africa contributed to the data synthesized in this review, we were not able to present data for all sub-regions of the continent or by country income category. Thirdly, as data included in this review were collected mainly in hospital settings as opposed to from community surveys, we were unable to stratify our results to urban versus rural areas to assess whether hygiene and sanitation affect the current epidemiology of HAV in Africa. Lastly, although trends in publication of the immunisation research is growing, a lot of research work in Africa still remains unpublished and access to such information is limited [58]. Regardless of these limitations, it is noteworthy to mention that the majority of included studies focused on hepatitis A data in childhood and adolescent populations, which may attest to the anecdotal evidence that children and adolescents are increasingly at risk for acute hepatitis A infection in Africa.

The results of this paper may be an over-estimate of HAV seroprevalence for the general population in Africa as those seeking private healthcare services were not included in this review. Populations seeking private healthcare services are more likely to be of higher social economic status. Higher social economic populations have access to optimal sanitation and are likely to show lower HAV seroprevalence although some may be vaccinated against the disease [27]. Furthermore, the extent of HAV vaccine use in the private sector of Africa is unknown. Future research should include populations seeking both private and private healthcare. Measurement of both IgG and IgM as immunological outcomes should be incorporated in future studies as well as details of the assay detection limits used. Additional missing data such as morbidity, co-morbidity and mortality due to hepatitis A disease should be a research priority. Collectively, complete and high-quality hepatitis A epidemiology data would allow for better pooling of results and meta-analyses. The review team also encourages future studies to incorporate mathematical modelling where the data permits as such an approach could possibly assist health policy decision-makers to better design hepatitis A control strategies in Africa.

## Conclusion

This systematic review aimed to generate up-to-date epidemiological data of hepatitis A in Africa with the aim of providing data to better inform hepatitis A public health control measures in the region. We successfully addressed the aim of the study although data on hospitalization, case fatality rates and co-morbidity was missing. With no current routine use of hepatitis A vaccines on the African continent, quality epidemiological data that is currently missing should be compiled and priority be given in re-assessing the current hepatitis A control strategies in the region to prevent possible disease outbreaks in the future.

## Additional file

Additional file 1: PRISMA checklist. (PDF 60 kb)

## Data Availability

The datasets generated and/or analysed during the current study are not publicly available due to ongoing research but are available from the corresponding author on reasonable request.

## Abbreviations

EPI: Expanded Programme on Immunisation
HAV: Hepatitis A virus
IgG: Immunoglobin class G antibodies
IgM: Immunoglobulin class M antibodies
LMICs: Low and Middle-Income Countries
MeSH: Medical Subject Headings
nRCT: non-Randomised Control Trial
PRISMA: Preferred Reporting Items for Systematic Reviews and Meta-analyses
RCT: Randomised Control Trial
RT-PCR: Real time PCR
SES: Socio-Economic Status
VPD: Vaccine Preventable Disease
WHO: World Health Organization
